# Real-time prediction of patient immune cell modulation during irreversible electroporation therapy

**DOI:** 10.1038/s41598-019-53974-w

**Published:** 2019-11-28

**Authors:** N. Beitel-White, R. C. G. Martin, Y. Li, R. M. Brock, I. C. Allen, R. V. Davalos

**Affiliations:** 1 Bioelectromechanical Systems Laboratory, Virginia Tech - Wake Forest University School of Biomedical Engineering and Sciences, Blacksburg, VA 24061 USA; 2 Department of Electrical and Computer Engineering, Virginia Tech, Blacksburg, VA 24061 USA; 3 Division of Surgical Oncology, Department of Surgery, School of Medicine, University of Louisville, Louisville, KY 40202 USA; 4 Translational Biology, Medicine, and Health Graduate Program, Virginia Tech, Blacksburg, VA 24061 USA; 5 Department of Biomedical Sciences and Pathology, Virginia-Maryland College of Veterinary Medicine, Blacksburg, VA 24061 USA

**Keywords:** Surgical oncology, Biomedical engineering

## Abstract

Immunotherapies have demonstrated limited efficacy in pancreatic ductal adenocarcinoma (PDAC) patients despite their success in treating other tumor types. This limitation is largely due to the relatively immunosuppressive environment surrounding the tumor. A focal ablative technique called irreversible electroporation (IRE) has been shown to modulate this environment, enhancing the efficacy of immunotherapy. One enhancing factor related to improved prognosis is a decrease in regulatory T cells (T_reg_). This decrease has been previously unpredictable for clinicians using IRE, who currently have limited real-time metrics for determining the activation of the patient’s immune response. Here, we report that larger overall changes in output current are correlated with larger decreases in T cell populations 24 hours post-treatment. This result suggests that clinicians can make real-time decisions regarding optimal follow-up therapy based on the range of output current delivered during treatment. This capability could maximize the immunomodulating effect of IRE in synergy with follow-up immunotherapy. Additionally, these results suggest that feedback from a preliminary IRE treatment of the local tumor may help inform clinicians regarding the timing and choice of subsequent therapies, such as resection, immunotherapy, chemotherapy, or follow-up thermal or non-thermal ablation.

## Introduction

Patients with pancreatic cancer have one of the most dismal prognoses of all cancer types with an approximate five-year survival rate of 9%^1^. The only curative option for patients with pancreatic ductal adenocarcinoma (PDAC) is surgical resection, yet most patients present with unresectable tumors due to the proximity of the tumor to critical structures or with metastatic disease^2,3^. If PDAC patients present with resectable disease, surgery is the preferred treatment option^4^. However, a significant portion of PDAC patients present with metastatic disease. Surgical resection of metastases has not been shown to improve survival, and thus has not been incorporated into the standard of care which consists of aggressive combinations of chemotherapies^5^. Borderline PDAC has been shown to respond to neoadjuvant therapy with the goal of converting the disease to resectable, leading to curative resection and adjuvant chemotherapy^4^. The primary adjuvant therapies in use for treatment of resectable PDAC are FOLFIRINOX regimens (5-Fluorouracil, Leucovorin, Irinotecan and Oxaliplatin) as well as Gemcitabine-based treatments. Immune-based therapies have recently gained attention due to their early clinical success in altering the course of disease in patients with previously untreatable cancers^6–8^. Unfortunately, the relatively immunosuppressive nature of pancreatic cancer hinders the delivery of immunotherapies in PDAC as compared with other malignant tumors. This immunosuppressive phenotype of pancreatic cancer derives from specialized immune cells, such as regulatory T (T_reg_) cells, which ultimately mask the tumor to evade the surveillance of immune system^9^, resulting in a reduced anti-cancer immune response. Immunotherapy options such as vaccination and checkpoint inhibitors have shown limited success due to the lack of immune cell infiltration to the tumor site and tumor antigen availability^10,11^. All of these barriers have led to disappointing clinical results, dose-related toxicity, and harsh combinatorial regimens.

**Figure 1:**
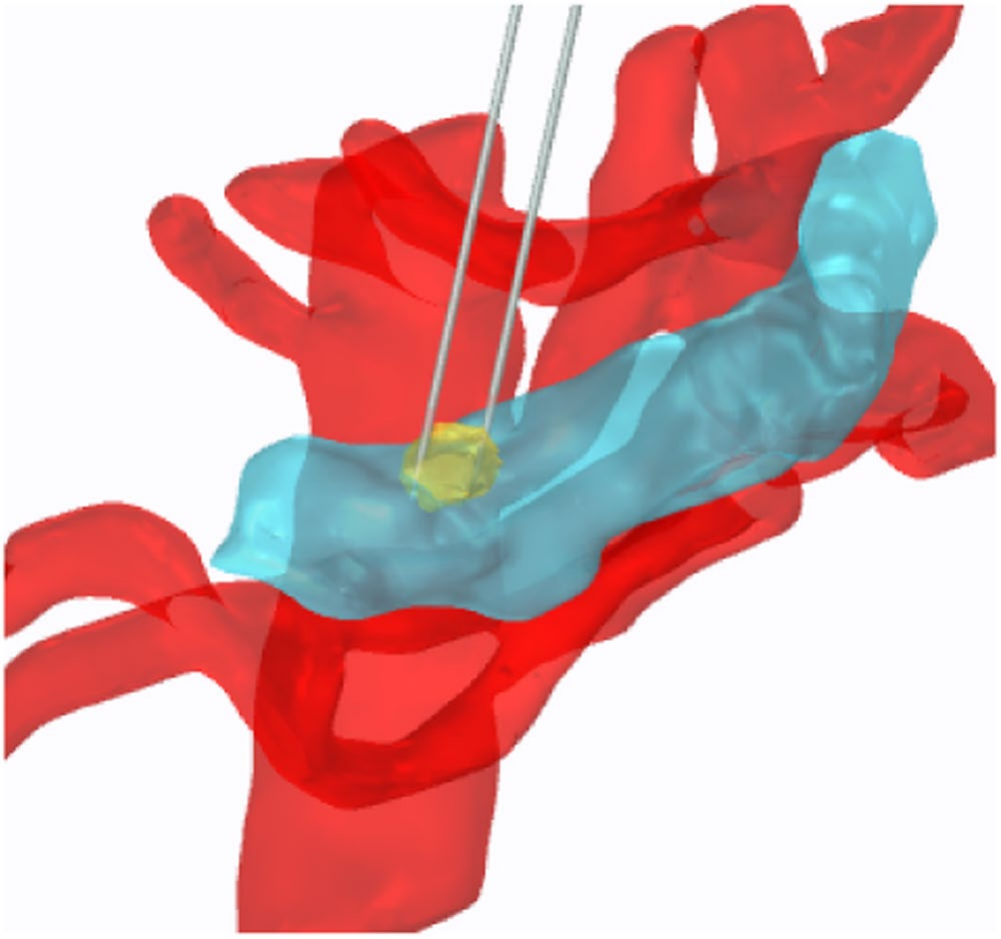
Schematic showing IRE treatment performed by directly inserting electrodes into the target treatment zone. A representative 3D reconstruction of a human pancreas (blue), tumor (yellow), and vasculature (red). Two electrodes (grey) are inserted into the tumor in this example. Pancreas and vasculature reconstructions were prepared using 3matic and Gmsh software using a pre-operative CT scan; electrodes and tumor mimic placed using COMSOL software (v. 4.1, Stockholm, Sweden).

Thermal ablative methods such as radiofrequency (RF) and microwave ablation not only locally destroy tumor tissue, but also modulate the immune response to overcome these barriers^12^. Additionally, electrochemotherapy (ECT) combined with calcium electroporation has been shown to induce systematic immune responses leading to regression of distal metastases^13^. Combinatorial treatments which pair ablative techniques with immunotherapies have also seen success^14^. However, the effects of thermal ablation result in protein denaturation that can significantly alter tumor neoantigens and attenuate immune system recognition. Irreversible electroporation (IRE) destroys cancerous cells by delivering short electric pulses through electrodes inserted directly into the targeted tumors (Fig. 1). Prior studies have shown that IRE induces cell death in targeted cancerous cells while maintaining the integrity of the stromal elements of the tissue^15,16^, enabling IRE to treat previously unresectable tumors while preserving nerves and major blood vessels in the treatment zone. When compared to thermal and cryo- techniques, IRE has been shown in an *in vitro* model to release the most antigens and proteins^17^, implying that IRE may optimally prime the immune response *in vivo*.

In addition to these advantages, IRE has also been shown to both protect against tumor rechallenge and reduce resistance to an immune checkpoint blockade in two different mouse models^18,19^. By using tumor disruptive approaches, the tumor cell can act as its own anti-cancer vaccine through the production of patient-specific tumor antigens associated with the ablated tissue^20^. The production of oncoantigens along with decreases in suppressive immune cell populations like T_reg_ cells in the ablation zone can lead to increased pro-inflammatory immune response following IRE^21^. This can potentially facilitate the destruction of both primary and metastatic tumors and prevent the likelihood of cancer recurrence following treatment. To date, IRE has been used in the clinic to treat human prostate, liver, and kidney tumors^22–24^. In locally advanced pancreatic cancer (LAPC) patients, IRE treatment has shown to nearly double the median survival when combined with chemotherapy and resection^25,26^. Since most types of chemotherapy have been shown to have a cytotoxic effect on immune cells^27^, patients demonstrating a decline in T_reg_ cells may benefit from the synergistic effects of IRE and immunotherapy.

Decreased circulating T_reg_ populations in the blood of pancreatic cancer patients undergoing chemotherapy have been shown to be associated with improved overall survival rates^28^. Recently, Scheffer *et al*. showed that IRE alleviates the immunosuppression induced by LAPC by reducing systemic T_reg_ populations and activating PD-1^+^ T cells^29^. These results suggest that IRE creates a transient reduction in immune system suppression which may be augmented by adjuvant immunotherapy. Here, we report that post-IRE T cell populations are correlated with the change in IRE current delivered to the local tumor. Specifically, an overall change of approximately 25 A of electrical current during IRE treatment resulted in a decrease in two T_reg_ sub-populations 24 hours post-IRE. Since current changes can be monitored in real time, future changes in T cell populations may be predicted during the IRE treatment. This ability to predict changes in the patient’s systemic immune system may improve treatment applications and equips clinicians to maximize post-treatment options.

## Results

### IRE induces changes in T cell populations which correlate with changes in electrical current

**Figure 2:**
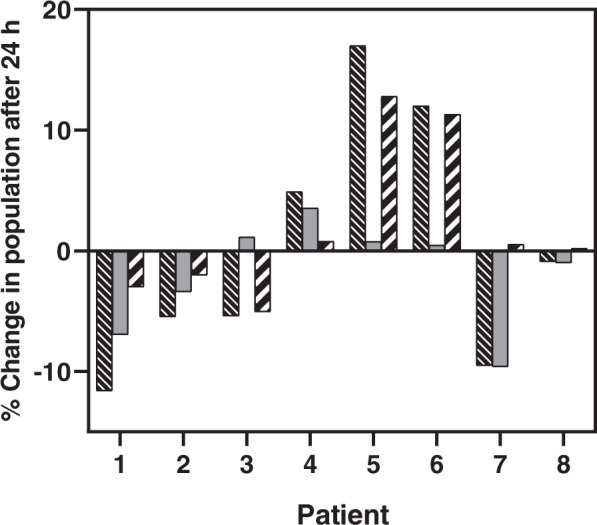
Percent change in T cell sub-populations CD4+ CD25+, CD4+ CD25+ FoxP3+, and CD4+ CD25+ FoxP3- differed across patients after 24 hours post-IRE. Populations were calculated as a subset of total CD4+ cells. Legend:  CD4+ CD25+,  CD4+ CD25+ FoxP3+, and  CD4+ CD25+ FoxP3−.

T cell sub-populations measured from eight patients before and 24 hours after IRE treatment show an array of responses (Fig. 2). Particularly, sub-populations CD4+ CD25+, CD4+ CD25+ FoxP3+, and CD4+ CD25+ FoxP3- were isolated from blood samples and counted using flow cytometry. Of the patients treated with IRE, 7 of 8 exhibited significant alterations in T_reg_ and CD4+ CD25+ FoxP3- sub-populations.

**Figure 3:**
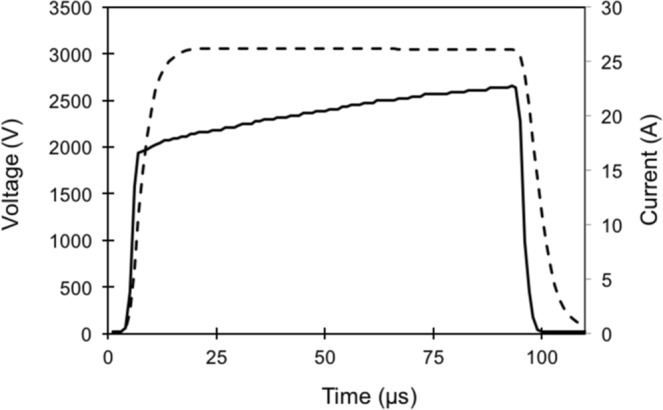
Waveforms captured from IRE generator enable analysis of changes in electrical current. Shown is a representative IRE pulse delivered using the Nanoknife® device. Current and voltage waveforms were saved in XML format for further analysis. The resistance value resulting from the applied voltage and current is displayed to the user during treatment. Legend: ---Voltage, — Current.

**Figure 4:**
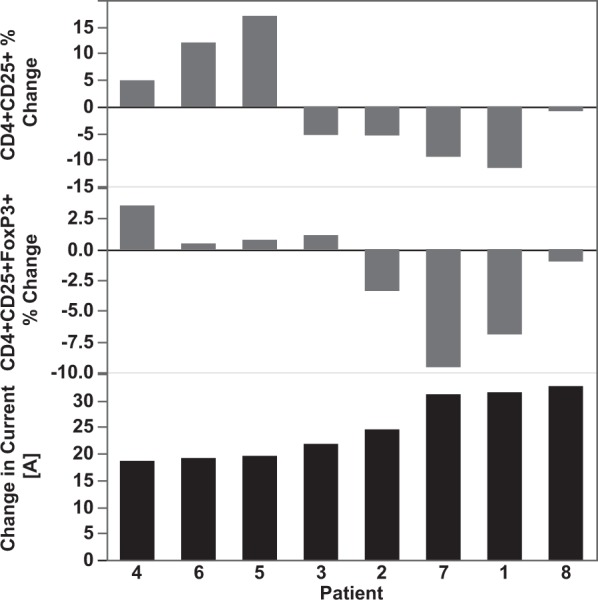
IRE patients exhibited an array of changes in T_reg_ cell levels and electrical current following IRE. Shown is the percent change in CD4+ CD25+ and CD4+ CD25+ FoxP3+ 24 hours after IRE. The changes are shown in order of increasing overall change in output current (lower-most panel) during the treatment in each patient. Populations were calculated as a subset of total CD4+ cells.

**Figure 5:**
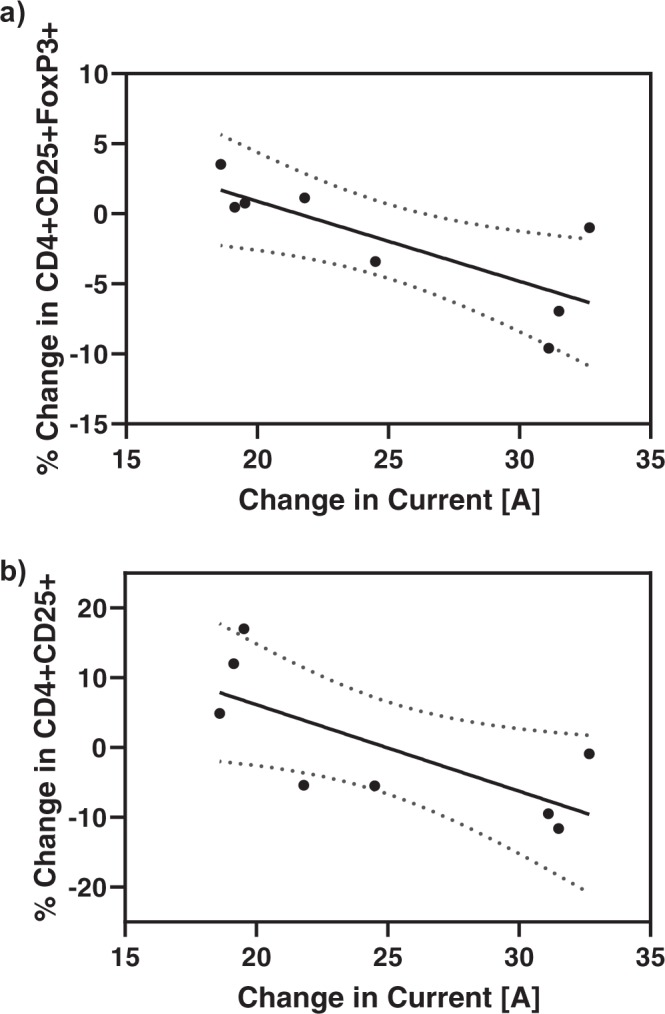
Changes in T_reg_ cell populations were found to linearly correlate with changes in electrical current. The change in (**a**) CD4+ CD25+ FoxP3+ and (**b**) CD4+ CD25+ after 24 hours following IRE decreases linearly with the change in current delivered during the IRE treatment for n = 8 patients. A linear regression was performed using Prism software (version 8.1.2, GraphPad Software, Inc., La Jolla, CA). The regression statistics were R^2^ = 0.5955, p = 0.0249 and R^2^ = 0.5268, p=0.0415 for (**a**) and (**b**), respectively.

Electrical current values were extracted from the Nanoknife® device, and the change in current was calculated as the difference between the maximum and minimum output current values reached during treatment (Fig. 3), regardless of probe pair. This analysis took place post-treatment on recorded data stored in XML format. The eight patients had an array of responses in overall output current changes, ranging from approximately 17 A to 30 A. Interestingly, the changes in T_reg_ populations were found to linearly correlate with changes in the delivered electrical current (Fig. 4). In particular, an increase in current value during IRE treatment caused decreases in CD4+CD25+ cells and CD4+ CD25+ FoxP3+ cells, but was not correlated with the change in CD4+ CD25+ FoxP3- cells. A linear regression was performed to test the effect of change in current on total CD4+ CD25+ cells and CD4+ CD25+ FoxP3+ cells (Fig. 5), and both were found to be statistically significant (p < 0.05).

### Patients with decreases in T_reg_ sub-populations exhibit prolonged survival

**Figure 6:**
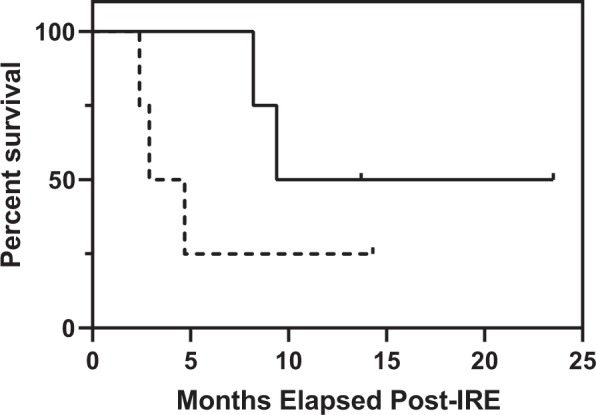
Survival curves show patients undergoing IRE treatment trend toward longer survival when they undergo an overall decrease in T cell populations 24 h post-treatment. Conversely, the subset of patients that did not exhibit a decrease in T cells trended toward shorter survival. A tick indicates the date of the final follow-up. Legend: — T cell Decrease, - - - T cell Increase or Same.

The overall survival of patients post-IRE treatment was examined in relation to the CD4+ CD25+ FoxP3+ sub-population (Fig. 6). The results show a trend towards increased survival following IRE for those patients with greater than a 2% decrease in T cells when compared with those who exhibited an increase or no change. Our results agree with previous studies in which low levels of T_reg_ cells are associated with improved prognosis^28,30,31^. However, we note that anti-tumor immune responses are multifactorial^32^. The IRE-induced immunomodulation in PDAC needs to be further studied to strengthen its predictive value.

## Discussion

This wide range of output current changes indicates that the content of the treatment zone varied from patient to patient, and the bulk tissue resistance changes differently in response to treatments with similar applied voltage and electrode spacing. Our results indicate that the degree to which the target tissue undergoes impedance changes affects cell populations related to the immune response in the ablation zone and the surrounding area. Electroporation induces pores in the membranes of cells in the target zone^33–35^, which may induce inflammation resulting in edema and the production of antigens. In turn, these changes reduce the impedance of the tissue resulting in an increased current output. Additionally, we hypothesize that while IRE retains the major proteins within the vasculature, the treatment may electroporate endothelial cells, which further promotes inflammation in the treatment site.

Following treatment of a patient with ablation, both an innate immune response and an adaptive immune response will occur. The adaptive immune response takes longer to activate (about 10–21 days) than the innate immune response, which peaks a few days (up to 72 hours or more) after treatment. The total response time for both the innate and adaptive immune responses are important factors when considering treatment options. An advantage of using a non-thermal ablation therapy such as IRE as a preliminary treatment of tumors is to give the patient’s innate and adaptive immune responses sufficient time to develop. Resection or subsequent therapies may have maximal impact when administered at least 10 days following IRE to allow the patient’s adaptive immune system to activate. However, one should note that excessive applied voltage and pulse number could lead to unnecessary Joule heating, which would counteract these advantages.

In our previous study, we found that T_reg_ sub-populations in peripheral blood were inversely affected by *in situ* IRE treatment in PDAC patients. Our current results indicate that IRE decreases T_reg_ populations, which may create an inflammatory response at the primary tumor site. Since systemic T_reg_ cell reduction has been previously shown to improve the prognosis of pancreatic cancer patients^25^, removal of these inhibitory cells could shift the tumor microenvironment from an anti-inflammatory state to be more favorable for anti-tumor immune system activation. During treatment, this shift may be predicted by monitoring the change in bulk tissue conductivity, which is reflected in the output current delivered through the electrodes. In practice, these results imply that the decline of the T_reg_ population can be predicted in real time using the IRE pulse delivery device itself, or an external device which measures impedance.

The relationship between change in current and changes in the patient’s immune cell populations, beyond just the T_reg_ cell populations, could also position IRE as a preliminary therapy that clinicians could use to screen patients for immunotherapy. We hypothesize that changes in these sub-populations may be predictive of adaptive immunity. For example, a low real-time change in output current may indicate an immunosupressive tumor environment which is less likely to shift. In this case, a clinician may choose not to follow with an immunotherapy due to the low response. Alternatively, an immune-responsive patient may benefit more from a delayed resection rather than immediately following IRE, giving the adaptive immune system a chance to fully develop in response to the treatment.

IRE offers a form of cell death which destroys tumor cells while preserving crucial proteins and antigens that serve to alert infiltrating immune cells of damage. The result that IRE modulates regulatory T cells systemically is a crucial first step towards assessing immunomodulation induced by IRE. Our present results are consistent with our previous studies which show modulation of these same T_reg_ sub-populations on day 3 and day 5 post-IRE^21^. Future work includes assessing additional time points beyond 24 h, and examining the predictive potential of electrical current changes. Ultimately, the ability to predict this immunomodulation *in vivo* provides clinicians with a metric they can use to optimize an IRE treatment and subsequent therapies, as well as determine which follow-up treatment may be optimal based on the predicted immune response during IRE. These particular thresholds for decreases in T_reg_ cells may possibly be limited to a particular set of pulse parameters; therefore, future work involves testing a wider variety of electrode and pulse configurations that may be used by clinicians. There also exists a balance between achieving adequate tumor coverage and applying excessive electrical energy resulting in Joule heating. In regards to the effects of IRE on the immune response, other systemic immune cells, tumor infiltrates such as B cells and natural killer cells^32^, as well as the presence of edema are of interest and would provide a more complete picture of the patient immune response both during treatment and post-IRE.

## Methods

Eight patients with stage III pancreatic cancer were treated with IRE via laparotomy. Prior to the electrode placement procedure, two-dimensional ultrasound imaging was used to check for metastatic disease and to confirm primary tumor size as previously described^25^. Ultrasound was used precisely during needle placement in order to bracket the primary tumor and safeguard proper needle placement. Patients were under appropriate paralytic and narcotic protocol. The first set of pulses consisted of 20 pulses per pair of probes that were used to assess local fibrosis and tissue resistance. The remainder of the treatment consisted of 100–220 pulses per probe-pair contingent on changes in resistance measured across each probe-pair. Pulse width was 90 *μ*s. Across all eight procedures, electrode spacing varied between approximately 1.5–2 cm, probe exposure varied between 1.0–1.5 cm, and applied voltage varied between 2550–3000 V. Either three or four electrodes were used and the pulse delivery device cycled between pulse pairs during the procedure. Depending on the size of the tumor, some treatments required the electrodes to be pulled back between 1–4 times in order to treat along the length of the tumor. In all cases, the pullback length was 0.5–1.0 cm.

Postoperative management of patients treated for pancreatic lesions with IRE was standard and followed guidelines for any type of pancreatic resection. These treatments were approved by and performed in accordance with the University of Louisville Institutional Review Board (02.0496 and 06.0326), and informed consent was obtained from all participants.

### Flow cytometry assay

Blood samples were drawn from the eight PDAC patients prior to and 24 h following IRE. Peripheral blood mononuclear cells (PBMCs) isolated and stored at −80 °C in RPMI media supplemented with 10% human serum albumin and 10% DMSO. To identify T_reg_ cells and FoxP3 subsets, isolated PBMCs were labeled using a FoxP3 kit (130-093-142, Miltenyi Biotech, Germany), then evaluated using FACSCalibur (BD Biosciences, San Jose, CA). Concentrations of CD4+ and CD25+ cells as well as T-regulatory levels were measured using a flow cytometer assay for PBMC in CD4+ cells. FoxP3 subset T_reg_s were identified as those stained with antibodies against FoxP3/CD25 and identified according to the expression of CD4+, CD25+ and FoxP3+ by fluorescence-activated cell sorting (FACS), and were evaluated at 1 × 10^5^/event using FACSCalibur (BD Biosciences, San Jose, CA). PBMC samples for each of the patients were analyzed simultaneously to decrease variability. FlowJo software (Ashland, OR) was used to analyze the data.

### Waveform analysis

Electrical current waveforms from the eight performed IRE treatments were collected from the IRE pulse generator (Fig. 3). Additionally, information about applied voltage, electrode number, and electrode spacing was extracted and compared. The total change in current as well as average current delivered were calculated using a MATLAB script (vR2016a, Mathworks Inc., Natick, MA). A linear regression was performed to examine the relationship between current and T_reg_ cell population changes using Prism software (version 8.1.2, GraphPad Software, Inc., La Jolla, CA). A statistical significance level of 0.05 was used for the analysis.

## Data Availability

The datasets generated and/or analyzed during the current study are available from the corresponding author on reasonable request.
